# Touch contamination and associated peritonitis in children undergoing maintenance peritoneal dialysis

**DOI:** 10.1007/s00467-026-07455-8

**Published:** 2026-07-10

**Authors:** Rebecca G. Same, Natalie K. Grills, Sreejata Dutta, Alicia M. Neu, Bradley A. Warady, Brandy Begin

**Affiliations:** 1 Department of Pediatrics, Perelman School of Medicine, University of Pennsylvania, Children’s Hospital of Philadelphia, Philadelphia, PA USA; 2 Children’s Hospital Association, Lenexa, KS USA; 3 Division of Pediatric Nephrology, Johns Hopkins University School of Medicine, Baltimore, MD USA; 4 Division of Pediatric Nephrology, Children’s Mercy Kansas City, Kansas City, MO USA; 5 Doernbecher Children’s Hospital at Oregon Health and Science University, Portland, OR USA

**Keywords:** Children, Touch contamination, Peritonitis, Maintenance peritoneal dialysis, Antibiotic prophylaxis

## Abstract

**Background:**

Infections are a leading cause of hospitalization and death in children receiving maintenance peritoneal dialysis (PD). Touch contamination is an established risk factor for development of peritonitis, but data regarding optimal management of touch contamination are limited.

**Methods:**

We conducted a retrospective cohort study using data submitted to the Standardizing Care to Improve Outcomes in Pediatric End Stage Kidney Disease (SCOPE) collaborative between 10/1/2011 and 7/31/2025 to evaluate touch contamination in children. Reported touch contamination events and their management, including administration of prophylactic antibiotics and transfer set changes, were evaluated. Univariate and multivariable generalized linear mixed modeling were used to assess relationships between demographic factors and episodes of touch contamination and between management of touch contamination events and temporally associated peritonitis. We also evaluated the microbiology of touch contamination-associated peritonitis and compared it to peritonitis not associated with touch contamination.

**Results:**

Of 2685 children included, 846 (32%) reported at least one touch contamination. In a multivariable model, younger age and non-Hispanic Black race were associated with increased rates of touch contamination. Administration of prophylactic antibiotics following touch contamination was associated with a lower prevalence of peritonitis (23% vs. 38%). Most episodes of touch contamination-associated peritonitis were Gram-positive (46%) or culture negative (24%).

**Conclusions:**

Touch contaminations are common in children undergoing maintenance PD. Administration of prophylactic antibiotics following touch contamination is associated with decreased risk of peritonitis.

**Graphical Abstract:**

A higher resolution version of the Graphical abstract is available as Supplementary information.

**Figure:**
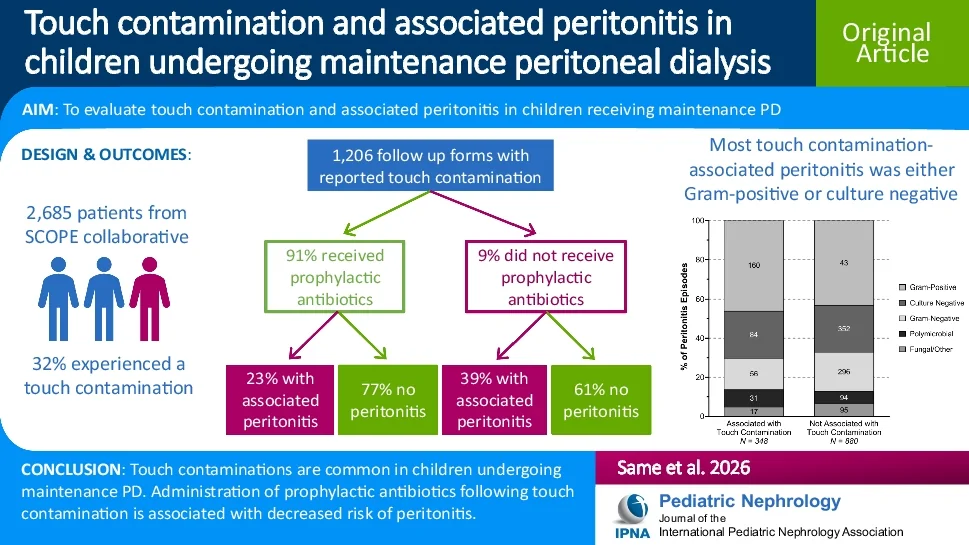

**Supplementary Information:**

The online version contains supplementary material available at https://doi.org/10.1007/s00467-026-07455-8.

## Introduction

For children receiving maintenance peritoneal dialysis (PD), infections are the most common cause of hospitalization and the second most common cause of death [1]. Touch contamination is an established risk factor for development of peritonitis, but data regarding optimal management of touch contamination are limited [2, 3]. Touch contaminations are classified as either dry contamination, defined as a contamination outside a closed PD system, such as connection distal to a closed clamp, or wet contamination, which occurs in an open system, when either dialysis fluid is infused after contamination or if the catheter administration set has been left open for an extended period of time [4]. One single-center study evaluated 548 episodes of touch contamination in adults and found that there were 17 episodes of peritonitis, all following wet contamination. They also found that the use of antibiotics following a wet contamination was associated with a significantly decreased risk for the development of peritonitis. Episodes of peritonitis associated with touch contamination were primarily culture-negative or caused by *Staphylococcus aureus* or coagulase-negative Staphylococci, i.e., bacteria typically found on the skin, with a minority caused by Gram-negative organisms [3].

This single study in adults informed both pediatric and adult guidelines about management of touch contamination in patients on PD, which recommend administration of prophylactic antibiotics for wet contaminations, but not for dry contamination [4, 5]. The optimal antibiotic choice has not been studied, but both guidelines suggest cefazolin based on the epidemiology identified in that single-center study.

No prior studies have comprehensively evaluated touch contamination or touch contamination-associated peritonitis in children receiving maintenance PD, which may differ from adults. Particularly in diapered children, it is unknown whether the organisms most associated with touch contamination are bacteria found on the skin vs. enteric Gram-negative organisms. There are also multiple approaches to touch contamination management, including administration of prophylactic antibiotics as well as changing or disinfecting the transfer set; the impact of these strategies on the risk of peritonitis, or the optimal antibiotics to use for prophylaxis, are unknown in children.

We conducted a retrospective analysis of data in the Standardizing Care to Improve Outcomes in Pediatric End Stage Kidney Disease (SCOPE) Collaborative to evaluate touch contamination in children receiving maintenance PD, with specific attention to the management of touch contamination, the risk for peritonitis, and the microbiology of touch contamination-associated peritonitis.

## Methods

### Study design

We conducted a retrospective cohort study of children receiving maintenance PD using data submitted to the SCOPE collaborative between 10/1/2011 and 7/31/2025. There were 57 pediatric dialysis centers participating in SCOPE and included in this study. The Institutional Review Boards at each center reviewed and approved the SCOPE protocol.

The study included data collected on the SCOPE follow up form, which is submitted monthly at the time of a routine clinical follow-up visit. The relevant questions on the form for the current analysis included whether touch contamination occurred since the prior follow-up visit and, if so, how many contaminations occurred (Supplemental Fig. 1). For each touch contamination identified, there are yes/no questions asking if (1) the transfer set was changed, (2) if not, was the transfer set disinfected, and (3) whether antibiotics were administered per International Society for Peritoneal Dialysis (ISPD) guidelines [4]. If antibiotics were not administered per guidelines, respondents are prompted to provide a reason using a dropdown menu with the following options: “no exchange occurred,” “clinic not notified,” “antibiotic given other than per ISPD guidelines,” or “other.” We also reviewed the follow up form question that asked whether the patient has “Urinary and/or stool incontinence requiring diaper?: YES/NO” as part of our data analysis.

### Definitions

Touch contamination events are identified on follow up forms that are submitted monthly for all patients with PD catheters. Follow up forms are completed at the time of a follow-up visit in the dialysis unit, which may occur at any time in the calendar month. Therefore, the interval between follow up forms, and the period during which a touch contamination may have occurred, will vary and could be as short as a few days, or as long as 8 weeks. The question on the follow up form asks if a touch contamination occurred since the last visit, but the exact date of the touch contamination event is not recorded. Conversely, peritonitis episodes are reported at the time of the infection. For example, if a follow up visit was performed on March 1st and the next follow up visit did not occur until April 30th, a touch contamination event that occurred on March 2nd would be reported on the April 30th follow up form. If an episode of peritonitis occurred and was reported on March 10th, the infection would be reported before the touch contamination, even though the touch contamination occurred first.

**Fig. 1:**
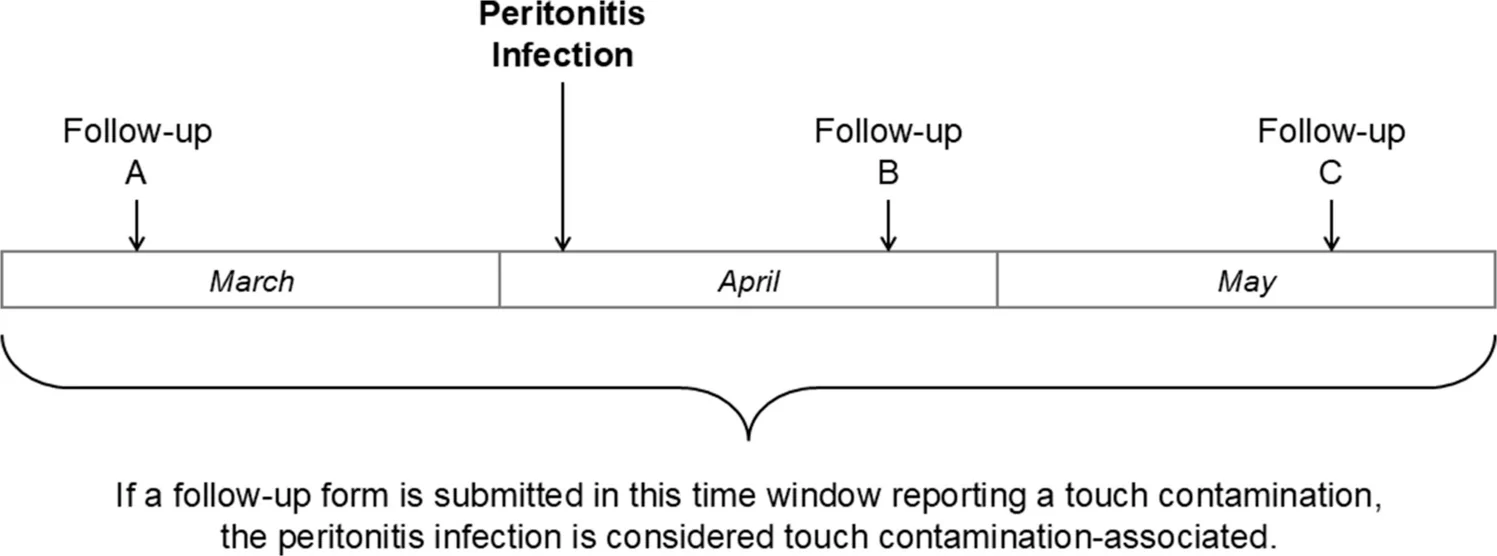
Touch contamination and peritonitis exposure window

To address this, we defined an episode of peritonitis as being associated with touch contamination if the infection form was submitted within a pre-defined touch contamination exposure window: the preceding, same, or following calendar month of a monthly follow-up form in which a touch contamination was reported (Fig. 1). Infections with no reported episodes of touch contamination in the exposure window were considered unrelated to touch contamination.

### Outcomes

Patient demographics, aside from age, were captured when patients first enrolled in SCOPE (either PD or hemodialysis registry). Age was calculated when patients initially enrolled in SCOPE PD.

Annualized touch contamination rates were calculated as the number of reported touch contaminations per 12 PD patient-months. Touch contamination rates were annualized due to the low number of touch contaminations.

### Statistical analysis

We evaluated demographic factors including age, sex, race, and primary renal diagnosis in children reporting ≥ 1 episode of touch contamination and compared them to children with no reported touch contaminations. For these comparisons, we employed chi-squared tests for categorical variables and Wilcoxon rank-sum tests for continuous variables. We summarized categorical variables using frequencies with percentages and continuous variables using medians with interquartile ranges (IQRs).

### Risk factors for touch contamination

To investigate touch contamination rates across patient characteristics, annualized touch contamination rates were modelled using a generalized linear mixed model that adjusted for race, sex, age at initial SCOPE PD enrollment, and primary renal diagnosis (congenital anomalies of the kidney and urinary tract (CAKUT) vs. not CAKUT/unknown). The model assumed an underlying negative binomial distribution and included a random effect to account for clustering of patients within hospitals. We used rate ratios (RRs) and 95% confidence intervals (CIs) to compare mean touch contamination rates across covariates in the model.

### Risk factors for development of peritonitis following a touch contamination event

To evaluate associations between management of touch contaminations and development of peritonitis, we conducted a subgroup analysis limited to follow up forms that only reported 1 touch contamination to eliminate forms with multiple touch contaminations that may have been managed differently. Additionally, we excluded forms that indicated “no exchange occurred” to limit the analysis to patients with wet contaminations. To separately assess whether (1) the administration of prophylactic antibiotics according to ISPD guidelines and (2) transfer set change or disinfection, were related to the prevalence of peritonitis within the exposure window, we performed chi-square tests. Information about administration of prophylactic antibiotics was available for the full study period. Questions about management of the transfer set were added to the follow up form on 8/1/2018.

### Microbiology of touch contamination-associated peritonitis

We evaluated the organisms causing touch contamination-associated peritonitis and compared that information to the organisms associated with episodes of peritonitis that were not associated with touch contamination, using chi-squared tests. Peritonitis episodes were classified as Gram-positive, Gram-negative, or fungal/other. Other organisms included mycobacteria or atypical bacteria. Peritonitis episodes were categorized as polymicrobial if more than one category of organism was identified (e.g., both a Gram-positive organism and a Gram-negative one) or as culture negative if no causative organism was identified. We also compared the microbiology in touch contamination-associated peritonitis in children who are diapered to those who are not. Children were considered diapered at the time of infection if the immediately preceding or following monthly follow up form indicated that the patient required a diaper.

All analyses were conducted using SAS version 9.4 (SAS Institute, Cary, NC), and *p* < 0.05 was considered statistically significant.

## Results

### Patient characteristics

A total of 45,983 forms, which represented 53,696 months of follow-up on 2685 children, were included in the analysis. Demographic characteristics of the cohort are shown in Table 1. At least one touch contamination event was reported for 846 of the 2685 patients (32%) during the study period. The median age at the time of touch contamination was 5 years (IQR 2, 14) and the median time from first PD enrollment to first reported touch contamination was 8 months (IQR 4, 16). The annualized peritonitis rate was higher among children with at least one reported touch contamination compared to those without any touch contaminations (median 0.3 (IQR 0, 0.83) vs. median 0 (IQR 0, 0.38), *p* < 0.001).

**Table 1:** Characteristics of children receiving peritoneal dialysis with and without reported touch contamination episodes

	Overall	≥ 1 Touch contamination	No reported touch contamination	***p***-value
Patients	2685	846 (31.5)	1839 (68.5)	
Patient months, *N* (%)	53,695.7	23,839.4 (44.4)	29,856.3 (55.6)	
Annualized peritonitis rate, median [IQR]	0 [0, 0.61]	0.30 [0, 0.83]	0 [0, 0.38]	< 0.001
Age at initial enrollment, *N* (%)				< 0.001
< 1 yr	483 (18)	217 (25.7)	266 (14.5)	
1–2 yrs	432 (16.1)	181 (21.4)	251 (13.6)	
3–5 yrs	219 (8.2)	66 (7.8)	153 (8.3)	
6–12 yrs	573 (21.3)	156 (18.4)	417 (22.7)	
13–17 yrs	786 (29.3)	188 (22.2)	598 (32.5)	
18 + yrs	192 (7.2)	38 (4.5)	154 (8.4)	
Sex, N (%)				0.042
Male	1529 (56.9)	506 (59.8)	1023 (55.6)	
Female	1156 (43.1)	340 (40.2)	816 (44.4)	
Race, N (%)				0.012
Non-Hispanic White	1245 (46.4)	392 (46.3)	853 (46.4)	
Non-Hispanic Black	402 (15)	153 (18.1)	249 (13.5)	
Hispanic	677 (25.2)	196 (23.2)	481 (26.2)	
Other/Unknown	361 (13.4)	105 (12.4)	256 (13.9)	
Primary renal diagnosis, N (%)				< 0.001
CAKUT	984 (36.6)	371 (43.9)	613 (33.3)	
GN	409 (15.2)	99 (11.7)	310 (16.9)	
PKD	140 (5.2)	52 (6.1)	88 (4.8)	
FSGS	368 (13.7)	92 (10.9)	276 (15)	
Ciliopathy	73 (2.7)	19 (2.2)	54 (2.9)	
Infarct	49 (1.8)	14 (1.7)	35 (1.9)	
Other/Unknown	662 (24.7)	199 (23.5)	463 (25.2)	

*IQR* interquartile range, *CAKUT* congenital anomalies of the kidney and urinary tract, *GN* glomerulonephritis, *PKD* polycystic kidney disease, *FSGS* focal segmental glomerulosclerosis

### Risk factors for touch contamination

**Fig. 2:**
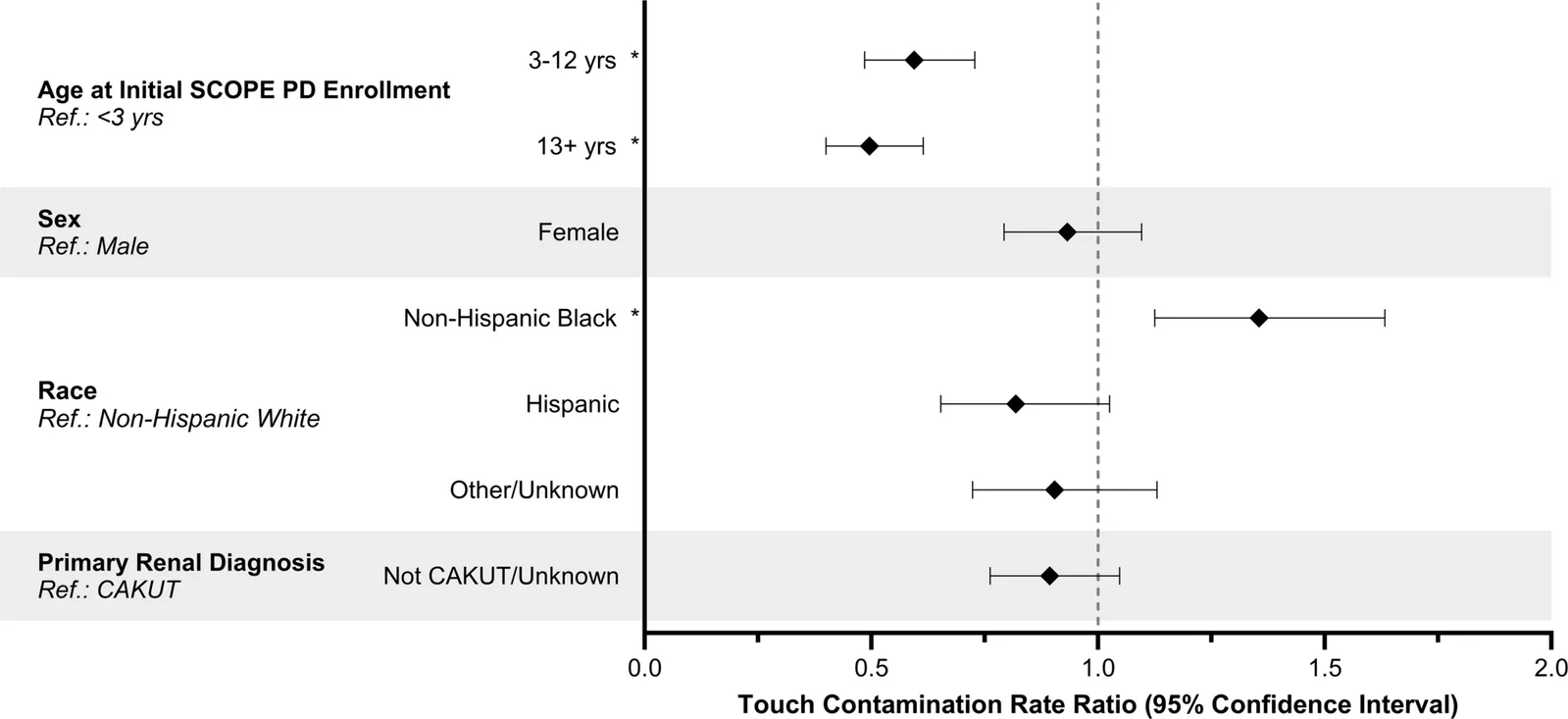
Forest plot of touch contamination rate ratios (RR) derived from generalized linear mixed model, adjusting for the following patient characteristics: age, sex, race, and primary renal diagnosis

The median annualized touch contamination rate among children with any reported touch contamination was 0.85 (IQR (0.51, 1.41)). Children who were younger at enrollment, particularly those aged <3years, were more likely to have a caregiver-reported touch contamination event, as were males, children with CAKUT, andnon-Hispanic Black children. In our multivariable model, age and race/ethnicity were found to be associated with theannualized touch contamination rate (Figure 2). The mean annualized touch contamination rate among children < 3 years old at the time of enrollment was 0.52 (95% CI (0.46, 0.59)). In comparison, the rates among patients aged 3–12 years and 13 years or older at enrollment were 40% and 50% lower, respectively (for 3–12 yrs, RR (95% CI): 0.595 (0.49, 0.73); for 13 + yrs, RR (95% CI): 0.50 (0.40, 0.61)) (Fig. 2). The touch contamination rate among non-Hispanic Black patients was 36% higher than the rate among non-Hispanic White patients (RR (95% CI): 1.36 (1.13, 1.63)). There were no other significant differences in touch contamination rates based on race or ethnicity, sex, or primary renal diagnosis.

### Risk factors for development of peritonitis following a touch contamination event

Excluding follow up forms indicating “no exchange occurred” (indicating dry contamination), there were a total of 1206 forms with only one instance of touch contamination reported (Table 2). Among these forms, prophylactic antibiotics according to the ISPD guidelines were administered following most touch contaminations (91%). The most common reason that ISPD guideline-concordant antibiotics were not given was that the clinic was not notified of the touch contamination, which occurred in 4% of all touch contamination events and 48% of those that did not receive antibiotics.

**Table 2:** Touch contamination management and associated episodes of peritonitis among follow up forms with only one touch contamination reported

	Overall	Touch contaminations with associated peritonitis	Touch contaminations without peritonitis	***p***-value
Follow up forms with 1 touch contamination	1206	297 (24.6)	909 (75.4)	
Received prophylactic antibiotics per ISPD guidelines				< 0.001
Yes	1079 (90.7)	248 (85.5)	831 (92.4)	
No	110 (9.3)	42 (14.5)	68 (7.6)	
If no antibiotics given per ISPD guidelines, why?				0.24
Clinic not notified	51 (48.1)	20 (48.8)	31 (47.7)	
Other antibiotics given	14 (13.2)	8 (19.5)	6 (9.2)	
Other	41 (38.7)	13 (31.7)	28 (43.1)	
Follow up forms with 1 touch contamination since 8/1/2018	610	141 (23.1)	469 (76.9)	
Transfer set changed				0.11
Yes	457 (75.8)	114 (80.9)	343 (74.2)	
No	146 (24.2)	27 (19.1)	119 (25.8)	
Transfer set changed				0.11
Yes	457 (75.8)	114 (80.9)	343 (74.2)	
No, but end of transfer set disinfected	82 (13.6)	11 (7.8)	71 (15.4)	
No, and end of transfer set not disinfected	38 (6.3)	11 (7.8)	27 (5.8)	
No, and unknown if end of transfer set disinfected	26 (4.3)	5 (3.5)	21 (4.5)	

*ISPD* International Society for Peritoneal Dialysis

One-quarter of the forms with only one reported touch contamination were associated with an episode of peritonitis during the exposure window. Receipt of prophylactic antibiotics following a touch contamination event was associated with a lower incidence of peritonitis, with 248/1079 (23%) of those who received prophylactic antibiotics experiencing peritonitis vs. 42/110 (38%) of those who did not (*p* < 0.001). Documentation that the transfer set was changed following a touch contamination event was present in 457 (76%) of 603 forms that included information about transfer set change. There was no significant association between transfer set change and the incidence of peritonitis related to a touch contamination episode.

### Microbiology of touch contamination-associated peritonitis

**Fig. 3:**
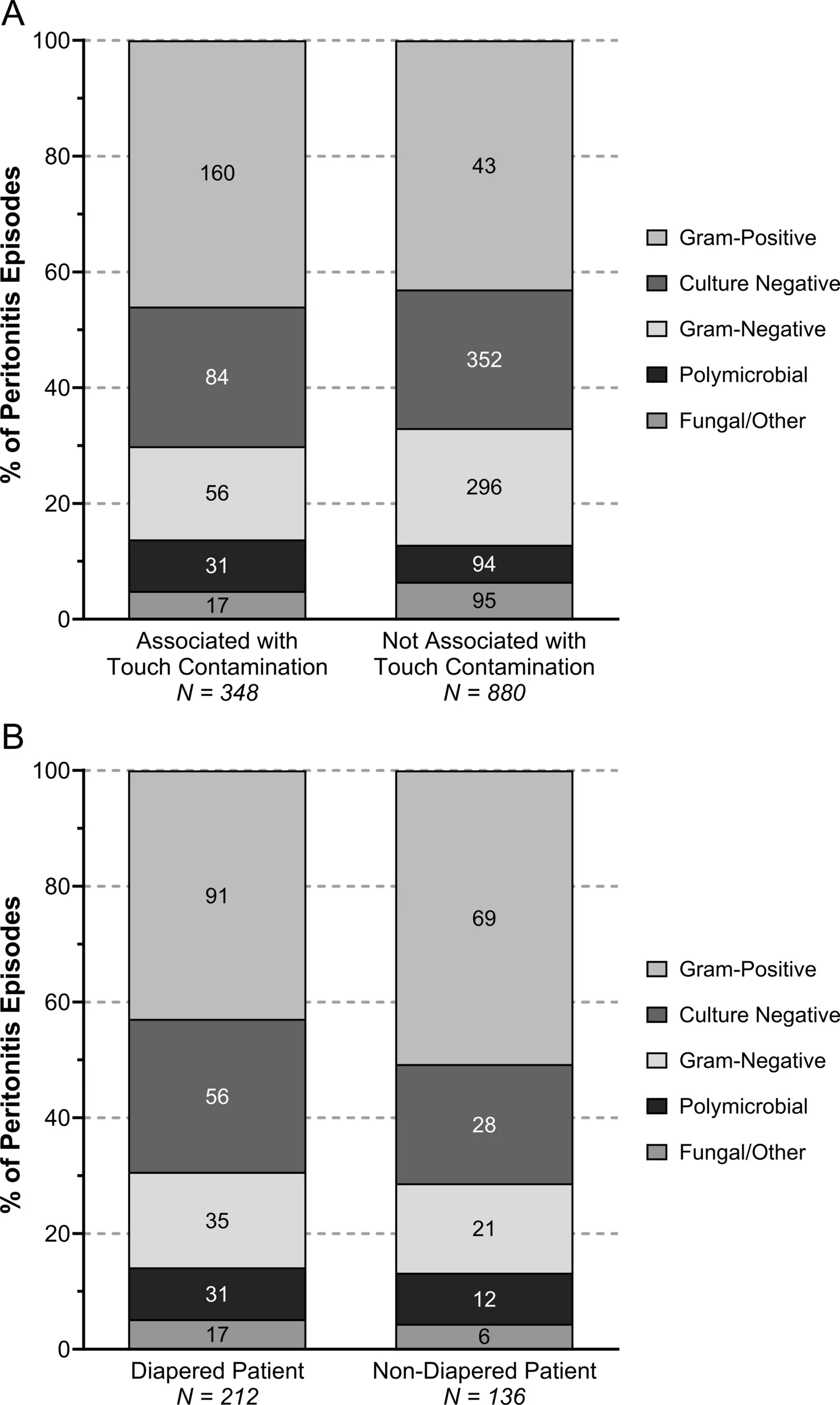
Microbiology of peritonitis episodes. **A** Peritonitis associated with an episode of touch contamination compared to peritonitis not associated with touch contamination. **B** Touch contamination-associated peritonitis in diapered patients compared to non-diapered patients

Most touch contamination-associated peritonitis episodes were either Gram-positive (46%) or culture negative (24%), with smaller percentages of Gram-negative (16%), polymicrobial (9%), and fungal/other (5%) peritonitis (Fig. 3). The most common Gram-positive pathogens were coagulase-negative Staphylococci (41%) and *Staphylococcus aureus* (21%).One-quarter of all *S. aureus* infections, both touch contamination-related and not, were methicillin-resistant, with MRSA making up a total of 3% of all episodes of peritonitis and 1% of those associated with touch contamination. *Pseudomonas* spp. was the most common Gram-negative organism, accounting for 20% of all Gram-negative infections, 4% of all infections, and 3% of those associated with touch contamination. The distribution of organisms was similar for patients with touch contamination-associated peritonitis and for peritonitis that was not associated with touch contamination (*p* = 0.09) and for diapered and non-diapered patients with touch contamination-associated peritonitis (*p* = 0.66).

## Discussion

In this multicenter cohort of children receiving maintenance PD, touch contamination was common, occurring in one third of children. One-quarter of forms with one reported touch contamination were associated with an episode of peritonitis within the exposure period. Prophylactic antibiotic administration was associated with a decreased risk of peritonitis. The microbiology of touch contamination-associated peritonitis, in both diapered and non-diapered patients, was similar to the microbiology of peritonitis for the overall cohort, predominantly Gram-positive and culture-negative. To our knowledge, this is the only multicenter study and the largest evaluation of touch contamination management and associated peritonitis in children or adults to date.

We found a much higher incidence of peritonitis associated with wet-touch contamination compared to the prior study in adults, where the incidence of peritonitis following wet contamination was 5.6% [3]. This may represent a real increased risk of peritonitis in children compared with adults, especially since we found that younger age was associated with an increased prevalence of touch contamination; however, it is more likely due to differences in definitions and measurement of peritonitis episodes. Our definitions were based on registry data and relied upon a wide exposure window, while the outcome in the prior study was peritonitis that developed within 72 h of touch contamination. The actual window from contamination to development of associated peritonitis is unknown and likely varies depending upon the virulence of the contaminating organism and the microbial burden of the contamination event. For example, the time to development of peritonitis may be short if there were a large inoculation event with a highly virulent organism, but it may be longer for lower inoculation events with less virulent organisms.

We observed that certain demographic factors were associated with an increased prevalence of touch contamination. Younger age was highly associated with an increased risk of touch contamination, which is not surprising given the technical challenges of administering PD to infants and toddlers. The impact of social determinants of health and systemic racism on children with stage 5 chronic kidney disease (CKD 5) is well documented and provides important context for our finding that the incidence of touch contamination varied across race, with the highest observed rate of touch contamination in non-Hispanic Black patients. Black children are more likely to have CKD 5, less likely to receive a kidney transplant, and have longer waitlist times for kidney transplant, increasing their time on dialysis and time at risk for touch contamination and infection [1, 6]. Additional research is needed to better understand and, most importantly, to address the underlying factors contributing to these disparities.

Most importantly, administration of prophylactic antibiotics per ISPD guidelines after a touch contamination event was associated with a reduced incidence of peritonitis. We were not able to evaluate or make recommendations for any specific antibiotic regimen, which is an important area for future investigation. However, the microbiology of touch contamination-associated peritonitis was similar to peritonitis not associated with a touch contamination event. Culture negative and Gram-positive infections predominate, even in diapered patients. This indicates that, as suggested in the ISPD guidelines, cefazolin is a reasonable option for prophylaxis in children after a touch contamination event [4]. This approach balances the benefit of prophylactic antibiotic administration for reduction in the risk of peritonitis with the risks associated with broad-spectrum antibiotic use, including the increased risk for fungal peritonitis as well as antibiotic resistance and antibiotic-associated adverse events [7, 8]. Of those children who did not receive prophylactic antibiotics after a wet contamination, the most common reason was that the clinic was not notified of the contamination event. Future work is needed to identify barriers to patients and caregivers reporting touch contamination events at the time of the event to allow for timely administration of antibiotic prophylaxis, especially given the demonstrated association between receipt of prophylaxis and the lower risk of associated peritonitis.

Our study has several limitations. First, our analysis was based on registry data and there may be variability in how individual centers classify data, though SCOPE provides training on all forms and data entry when centers join and as requested thereafter to promote accurate data entry. Due to the structure of the data forms, we did not know the exact dates that touch contamination occurred, so we used a relatively long exposure window to identify touch contamination-associated peritonitis. Because the actual timing from contamination to development of infection is unknown, it is not clear what the gold standard exposure window should be and it is likely that shorter windows underestimate the association of touch contamination with peritonitis. Additionally, because we relied on reporting of touch contamination events on monthly forms, we likely underestimated the incidence of touch contamination, which is not always reported by patients or caregivers to dialysis centers. Because we did not begin collecting data about transfer set changes until several years into the study period, we also had a relatively small number of forms with information about touch contamination events and transfer set changes, which may not have been adequate to demonstrate an association between the procedure and risk of infection.

In conclusion, touch contamination events occurred in approximately one-third of children receiving maintenance PD in this multicenter cohort. In a multivariable model, demographic risk factors for touch contamination included younger age and non-Hispanic Black race. Most episodes of peritonitis, including those associated with touch contamination in both diapered and non-diapered patients, were Gram-positive or culture negative, which supports the use of cefazolin for prophylaxis after touch contamination. Administration of prophylactic antibiotics was associated with a lower prevalence of touch contamination-associated peritonitis, a finding that should be emphasized to families/patients during training for home PD to encourage timely reporting of contamination events.

## Supplementary Information

Below is the link to the electronic supplementary material.

ESM1 (DOCX 156 KB)

Graphical Abstract (PPTX 142 KB)
