# Supplementary material for: Touch contamination and associated peritonitis in children undergoing maintenance peritoneal dialysis
Source: Pediatr Nephrol. 2026 Jul 10;41(12):4589–96. doi: 10.1007/s00467-026-07455-8 (PMC13672569; doi:10.1007/s00467-026-07455-8)
Supplement: Supplementary file 1 [file 467_2026_7455_MOESM1_ESM.docx]

**Supplemental Figure 1.** SCOPE Peritoneal Dialysis Follow up Form Touch Contamination Reporting Section.
